# Hepatitis C virus infection contributes to impregnation of markers of immune inhibition: potential preludes underlying viral latency and persistence

**DOI:** 10.1186/1471-2334-14-S3-P3

**Published:** 2014-05-27

**Authors:** Muttiah Barathan, Mohamed Rosmawati, Jamuna Vadivelu, Marie Larsson, Vijayakumar Velu, Saeidi Alireza, Li Yen Chang, Esaki Muthu Shankar

**Affiliations:** 1 Tropical Infectious Disease Research and Education Center (TIDREC), Department of Medical Microbiology, Faculty of Medicine, University of Malaya, Kuala Lumpur, Malaysia; 2 Department of Medicine, Faculty of Medicine, University of Malaya, Kuala Lumpur, Malaysia; 3 Department of Clinical and Experimental Medicine, Division of Molecular Virology, Laboratory 1, Level 13, Linköping University, 58185 Linköping, Sweden; 4 Department of Microbiology and Immunology, Emory Vaccine Center, 954 Gatewood Road, Atlanta, Georgia, USA

## Background

Hepatitis C virus (HCV) represents one of the persistent viral infections afflicting humankind, and a significant proportion of chronic HCV disease progresses over time through liver fibrosis, cirrhosis and hepatocellular carcinoma (HCC). One potential mechanism underlying the chronic disease is believed to be viral escape from immune surveillance via upregulation of inhibitory molecules on immune cells by HCV. We investigated the diverse expression of various inhibitory molecules in PBMCs of healthy non-HCV controls and chronically HCV infected patients.

## Methods

The expression of inhibitory molecules on PBMCs was investigated in chronic HCV infected patients relative to healthy non-HCV controls using standard immunological and molecular methods. The serum levels of indoleamine 2, 3 deoxygenase (IDO) and cyclooxygenase-2 (COX-2) were also investigated.

## Results

The gene expression profile of chronically HCV infected patients was significantly different from control individuals. Our results showed upregulation of TIM-3 (*p*≤0.01), PD-1 (*p*≤0.01), FOXP-3 (*p*≤0.01), BLIMP-1 (*p*≤0.01), CD160 (*p*≤0.01), CTLA-4 (*p*≤0.01), TRAIL (*p*≤0.01), BTLA (*p*≤0.01) and LAG-3 (*p*≤0.01) with fold change of 1.3, 0.4, 14.6, 0.87, 6.6, 0.4, 14.7, 10.9 and 2.5 respectively in chronically HCV infected patients. The plasma IDO and COX-2 levels were significantly higher (*p*=0.001) in chronically HCV infected subjects relative to healthy control.

## Conclusion

The upregulation of inhibitory molecules on PBMCs in chronically HCV infected patients suggest the contribution of these molecules to immune cells impairment in HCV infection. Viral persistence and eventual progression following potential evasion of the host immune armory via viral impregnation of inhibitory immune biosignatures in HCV disease pathogenesis warrants further elucidation.

